# Prepatellar Fibroblastic Sarcoma: A Real Diagnostic and Therapeutic Challenge

**DOI:** 10.7759/cureus.92990

**Published:** 2025-09-23

**Authors:** Achraf Tebbaa El Hassali, Taha El Aissaoui, Adnane Lachkar, Hicham Yacoubi, Najib Abdeljaouad

**Affiliations:** 1 Orthopedics and Traumatology, Mohammed VI University Hospital, Faculty of Medicine and Pharmacy of Oujda, Mohammed I University, Oujda, MAR

**Keywords:** fibroblastic, gastrocnemius flap, myxo-inflammatoy, patella, sarcoma, tumor

## Abstract

Myxoinflammatory fibroblastic sarcoma is a rare, low-grade tumor. It is an aggressive tumor of soft tissue or bone that can affect any part of the body. It is often located in the soft tissues of the distal extremities of middle-aged adults.

We report the case of a patient with myxo-inflammatory fibroblastic sarcoma surrounding the patellar tendon. The interesting aspect of our case lies in the rarity of the tumor and its particular location, constituting a real diagnostic and therapeutic challenge. In addition, we compared our case to recent literature.

## Introduction

Sarcoma is a malignant tumor of soft tissue or bone that can affect any part of the body. Myxoinflammatory fibroblastic sarcoma (MIFS) is a rare, low-grade soft tissue tumor that typically occurs in the distal extremities of middle-aged adults. It is located in the fingers and hand in nearly 75% of reported cases [1].

Histologically, MIFS originates from fibroblasts--the cells responsible for producing connective tissue. It is characterized by a mixture of fleshy spindle and epithelioid cells, inflammatory infiltrates, and mucin deposits within a fibrosclerotic stroma [2].

Fibroblastic sarcoma is considered an ultra-rare tumor, with a prevalence of fewer than 2 cases per 100,000 people. It can be misdiagnosed as a benign lesion during clinical and radiological examinations, potentially leading to delayed treatment and significant morbidity [3]. Additionally, secondary fibrosarcomas of the bone can arise in the context of preexisting conditions such as bone infarcts, chronic osteomyelitis, Paget’s disease, or previously irradiated tissues [4].

We report the case of a patient with MIFS involving the region surrounding the patellar tendon. The significance of this case lies in the rarity of the tumor and its unusual anatomical location, which presents a true diagnostic and therapeutic challenge.

## Case presentation

The patient was a 68-year-old male farmer with a medical history of cataract surgery performed 10 years ago and chronic smoking, which he ceased a decade ago. There was no notable family medical history.

He presented for consultation due to the gradual onset, over the course of three months, of a mass in the right knee. The mass was painful and progressively increased in size. The patient reported no fever, weight loss, or night sweats. The pain was moderate in intensity, intermittent, and inflammatory in nature. Despite being able to walk, the patient experienced a limited range of motion due to the size of the mass and the associated pain. This restricted mobility led to a temporary suspension of his professional activities.

On initial clinical examination, the patient was conscious and hemodynamically and respiratorily stable. Vital signs were as follows: blood pressure 136/77 mmHg, respiratory rate 20 breaths per minute, heart rate 78 beats per minute, capillary blood glucose 0.98 g/L, and body temperature 37 °C.

**Figure 1:**
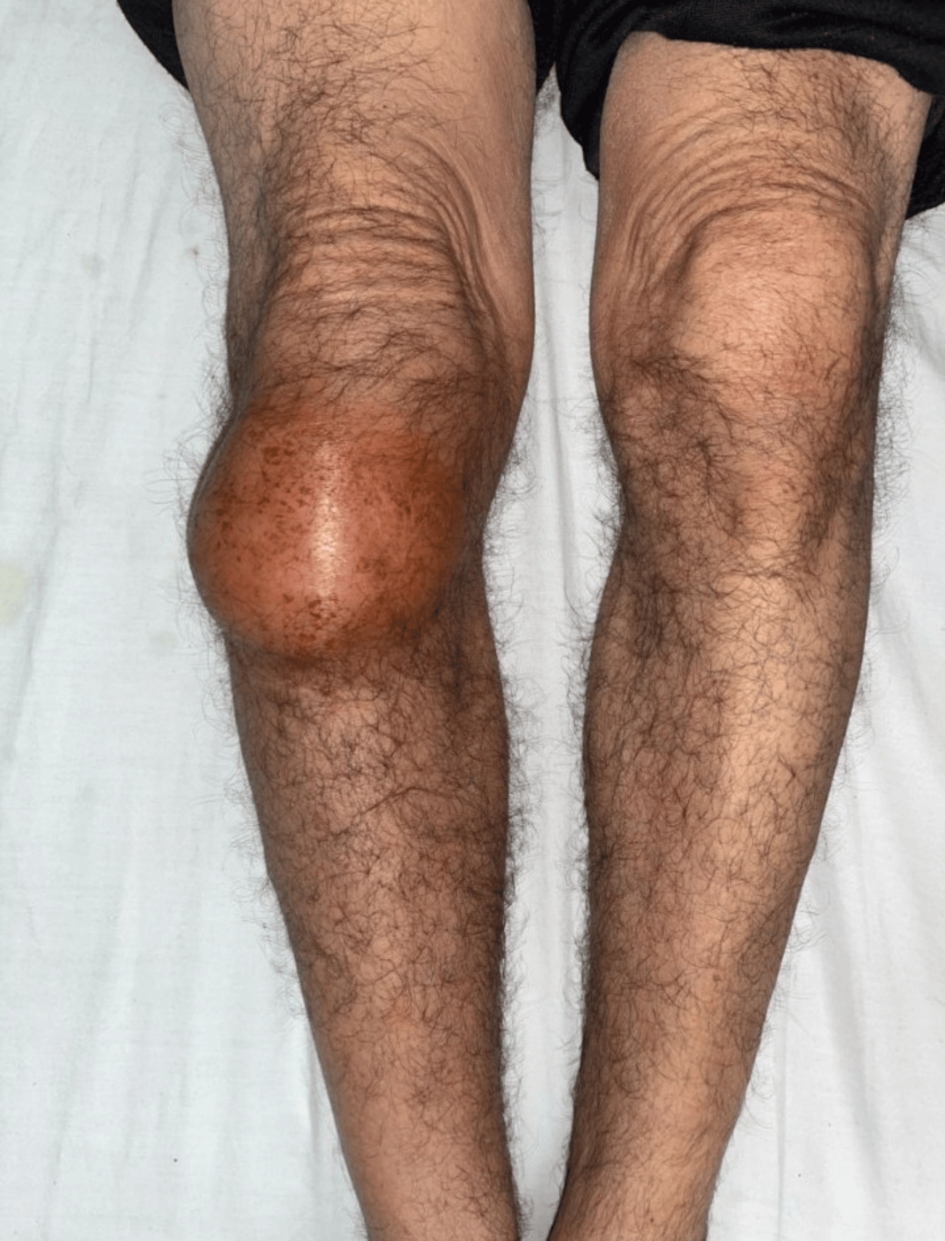
Image showing the clinical appearance of our patient's knee

The patient exhibited a limp while walking. Examination of the anterior surface of the right knee revealed a warm, painful, and mobile swelling on palpation. The mass was firm in consistency and caused a slight limitation of movement due to pain. Vascular and neurological examinations of the right lower limb were normal, and no lymphadenopathy was detected. The remainder of the clinical examination was unremarkable (Figure 1).

The patient was hospitalized and underwent a comprehensive laboratory evaluation, which returned normal results (Table 1).

**Table 1:** The biological assessment of our patient

Parameters	Patient’s value	Normal values
White blood cells	8600 /μl	4000–10.000/μl
Hemoglobin	14 gd/l	12–16 gd/l
Platelet count	280.000 /μl	150.000–40.000/μl
Urea	0.22 g/l	0.15–0.39 g/l
Creatinine	9.20 mg/l	5.7–11.1 mg/l
C-reactive protein	1.2mg/l	<5mg/l
Erythrocyte sedimentation rate	5 mm/h	<15 mm/h
Lactate dehydrogenase	160 UI/l	125-220 UI/l
Gamma-glutamyl transferase	20 UI/l	9–36 IU/l
Alkaline phosphatase	98 UL/l	40-150 UI/l

**Figure 2:**
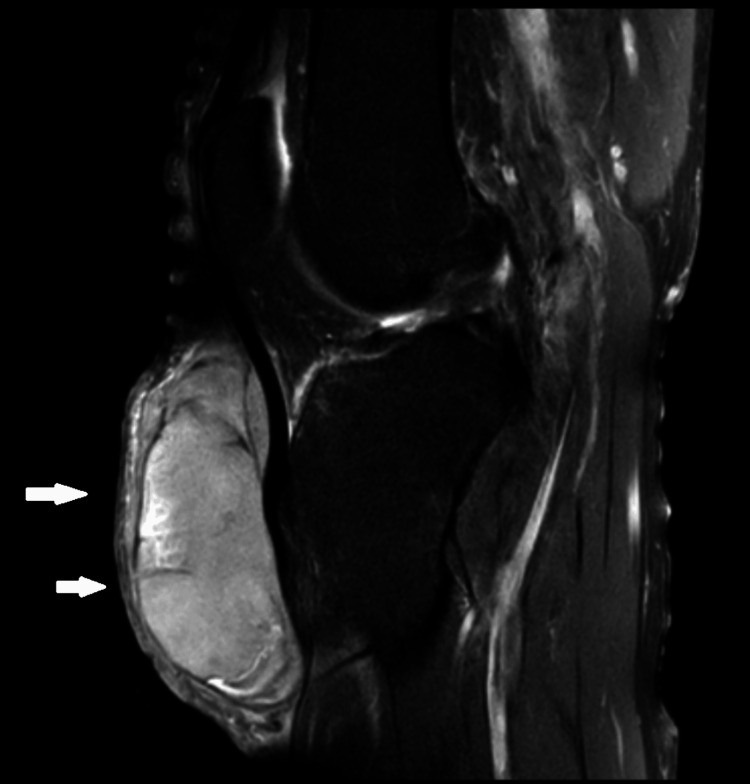
Sagittal MRI section showing the tumor mass This sagittal section shows a well-defined mass, with regular contours, in intermediate T2 hypersignal, in T1 hyposignal, and in diffusion hypersignal, which enhances heterogeneously after a gadolinium injection. The arrows show the location of the mass in the subcutaneous soft tissue of the right knee.

**Figure 3:**
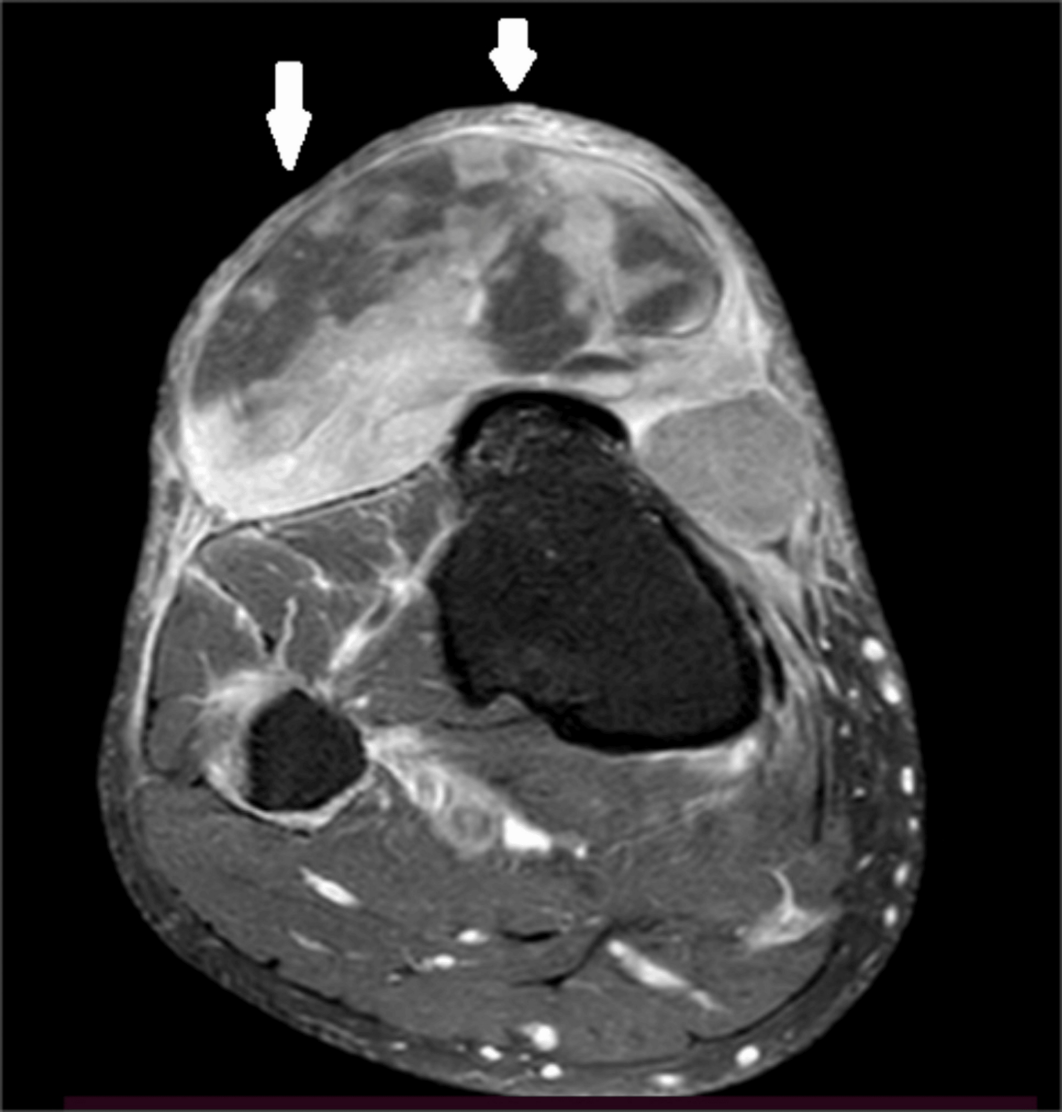
MRI image of the patient; axial section This axial section in the T2 sequence shows the multilobulated appearance of the mass, as well as the necrosis in spontaneous hypersignal, as shown by the arrows.

Standard anteroposterior and lateral X-rays of the right knee and leg were performed, followed by magnetic resonance imaging (MRI). MRI revealed a large soft tissue mass involving the right knee and leg, with heterogeneous signal characteristics of the subcutaneous soft tissues. The mass measured approximately 7.5 × 2.8 × 8.1 cm and contained areas of necrosis suggestive of a sarcomatous origin. Additionally, the MRI identified a grade II horizontal tear of the posterior horn of the medial meniscus and a thin layer of joint effusion (Figures 2-3).

After seven days of hospitalization, the patient underwent a percutaneous biopsy. Histological analysis of the specimen was consistent with a high-grade pleomorphic sarcoma (Grade III according to the FNCLCC (Fédération Nationale des Centres de Lutte Contre le Cancer) classification) (Figure 3).

Microscopic examination revealed a markedly atypical, highly cellular proliferation arranged in diffuse sheets. The tumor cells were predominantly spindle-shaped, with pleomorphic and hyperchromatic nuclei, and numerous mitotic figures, including atypical forms. The pathologist noted that the quality of the tissue fragments was moderate.

As part of the metastatic work-up, a cerebro-thoraco-abdomino-pelvic CT scan was performed and revealed no evidence of secondary lesions.

**Figure 4:**
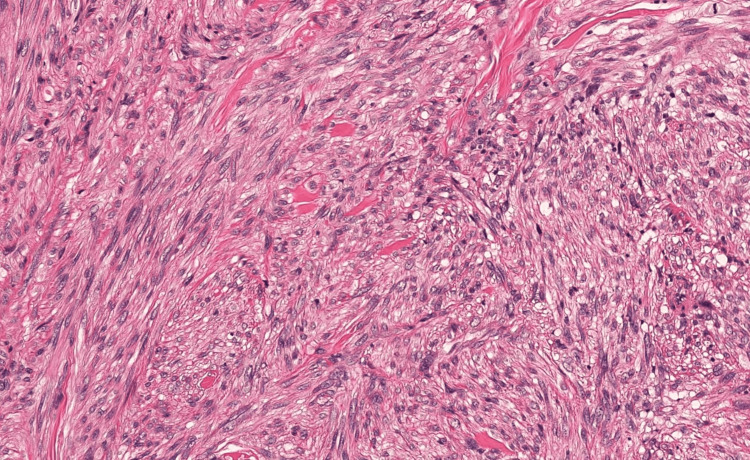
Histopathological image of the tumor mass Analysis of several fragments measuring 0.9*0.7*0.6 cm included in a single block with several cutting levels showed malignant tumor proliferation arranged in diffuse sheets of highly atypical cells, with segmented, pleomorphic nuclei in anisocariasis, with irregular contours and heterogeneous chromatin. The cytoplasm is abundant and often eosinophilic in appearance and shows anisocytosis (score 3). We also note numerous mitotic figures estimated at 20 mitoses/10 high-magnification fields (score 3), sometimes with an atypical appearance. The stroma is fibro-inflammatory with an absence of vascular emboli and perineural sheathing.

The surgical management of this patient consisted of tumor resection with adequate margins, including a partial patellectomy. The oncologic surgery was followed by reconstruction of the patellar tendon and the creation of a gastrocnemius rotational flap with a skin graft, performed in close collaboration with the plastic and reconstructive surgery team (Figure 4).

**Figure 5:**
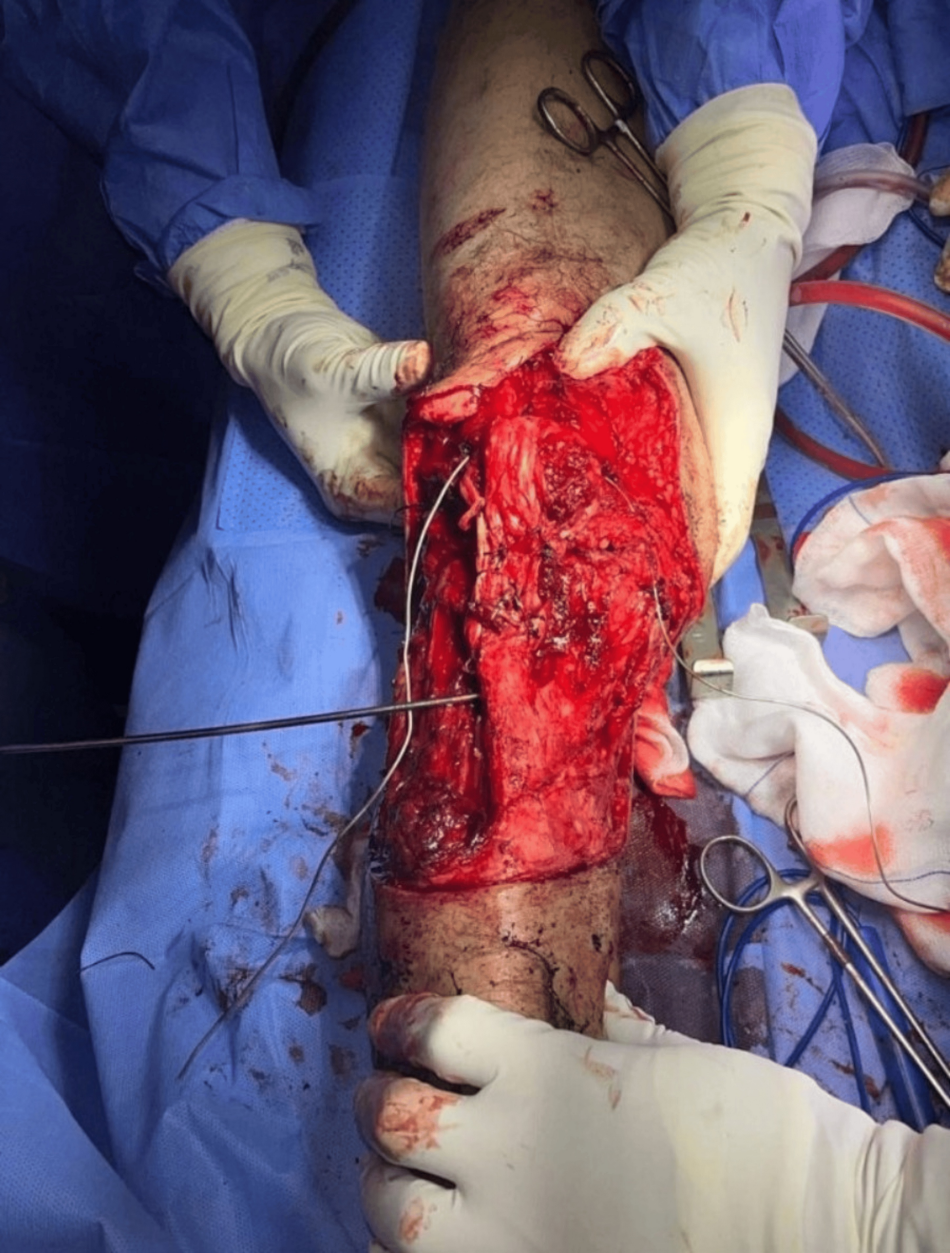
Surgical image showing tumor resection

**Figure 6:**
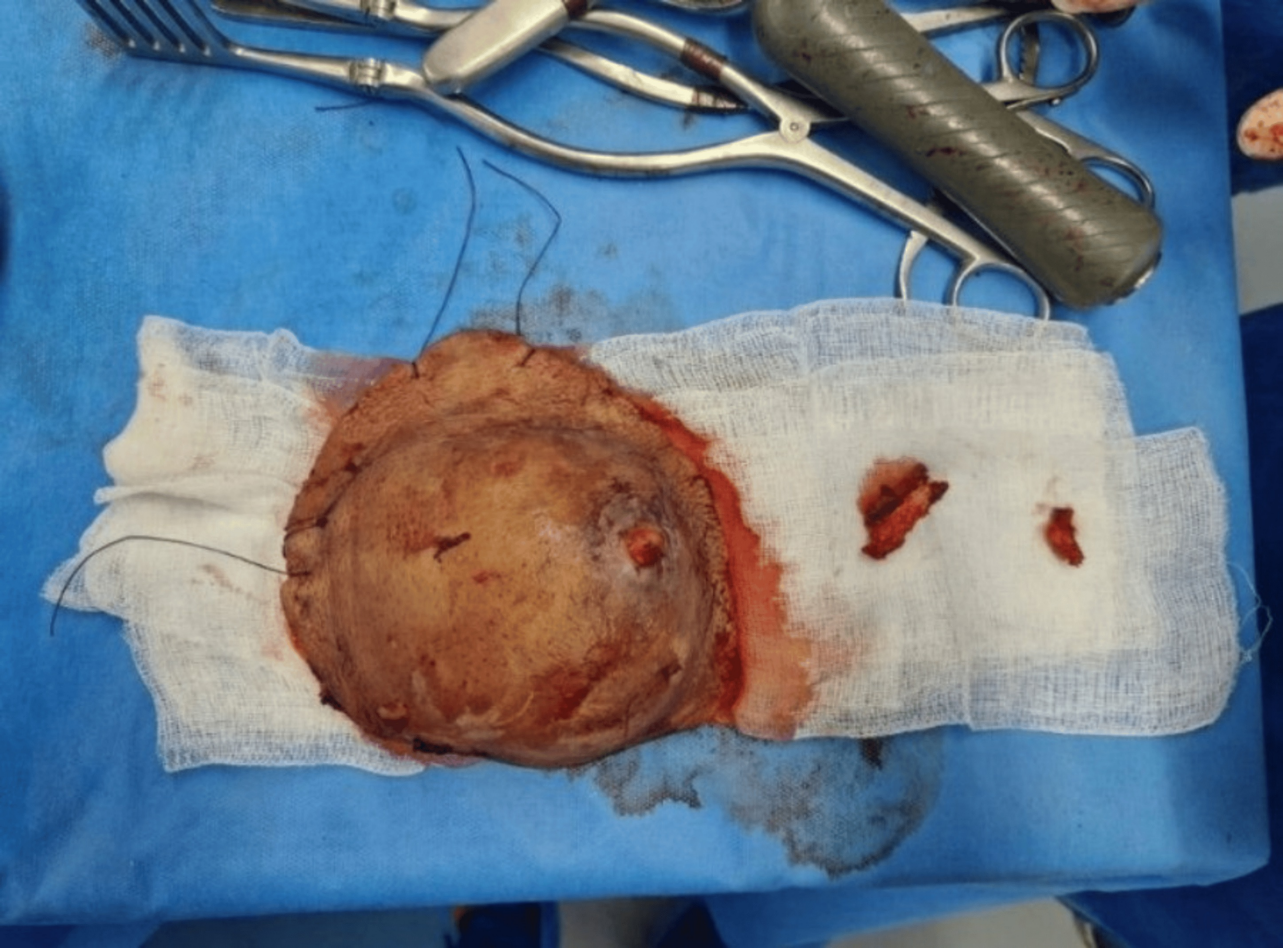
The surgical specimen The surgical specimen was located by a single thread at the upper pole and two threads at the medial part.

Specifically, the procedure involved a partial patellectomy (removal of the tip of the patella), followed by excision of a portion of the anterior cortex of the proximal tibia. Additionally, part of the pes anserinus tendons was resected to ensure clear surgical margins. The surgical specimen was marked with a single suture at the superior pole and two sutures at the medial aspect (Figure 5). Figure 6 shows the surgical specimen.

**Figure 7:**
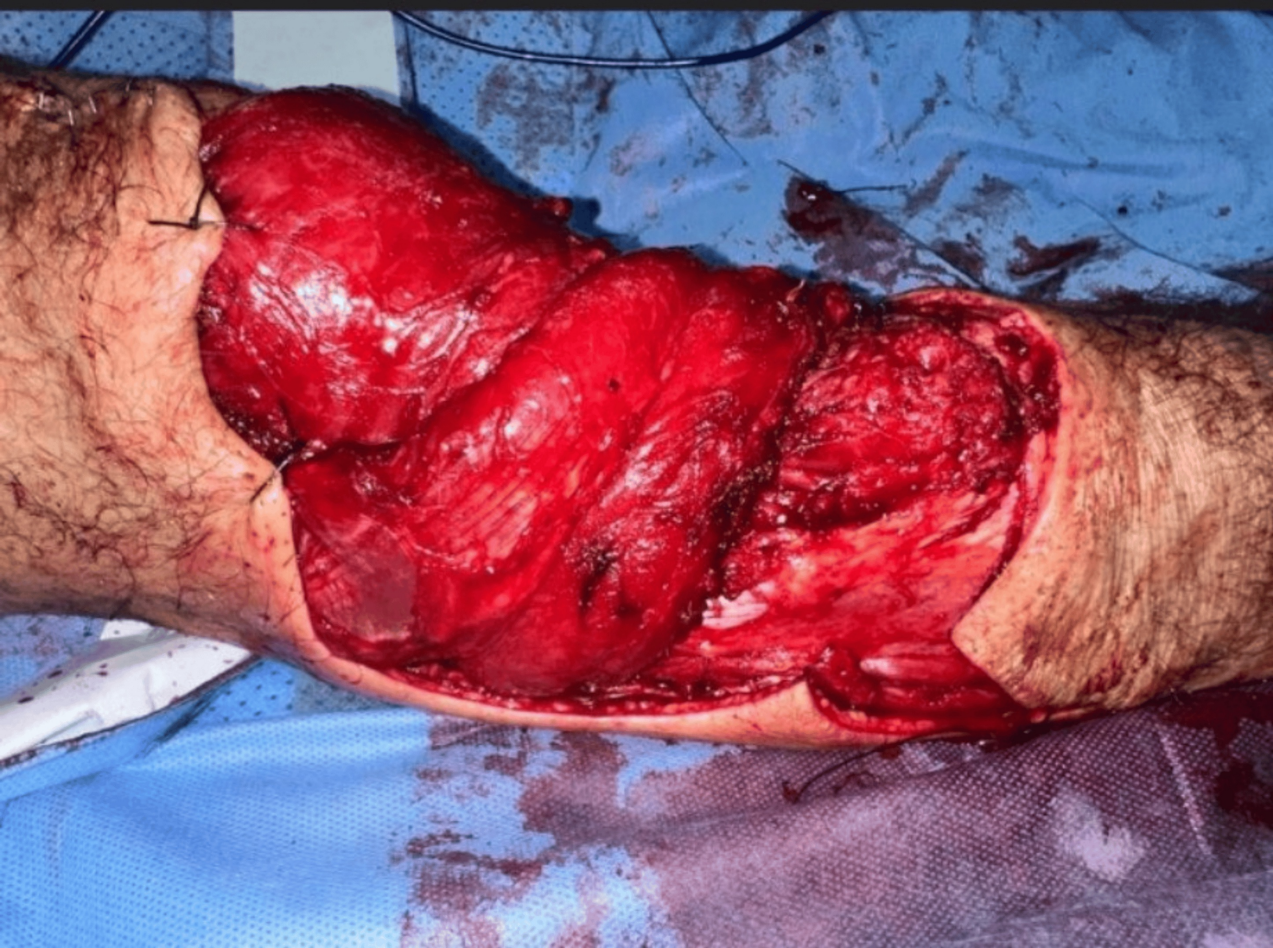
Operative image showing the gastrocnemius rotation flap

**Figure 8:**
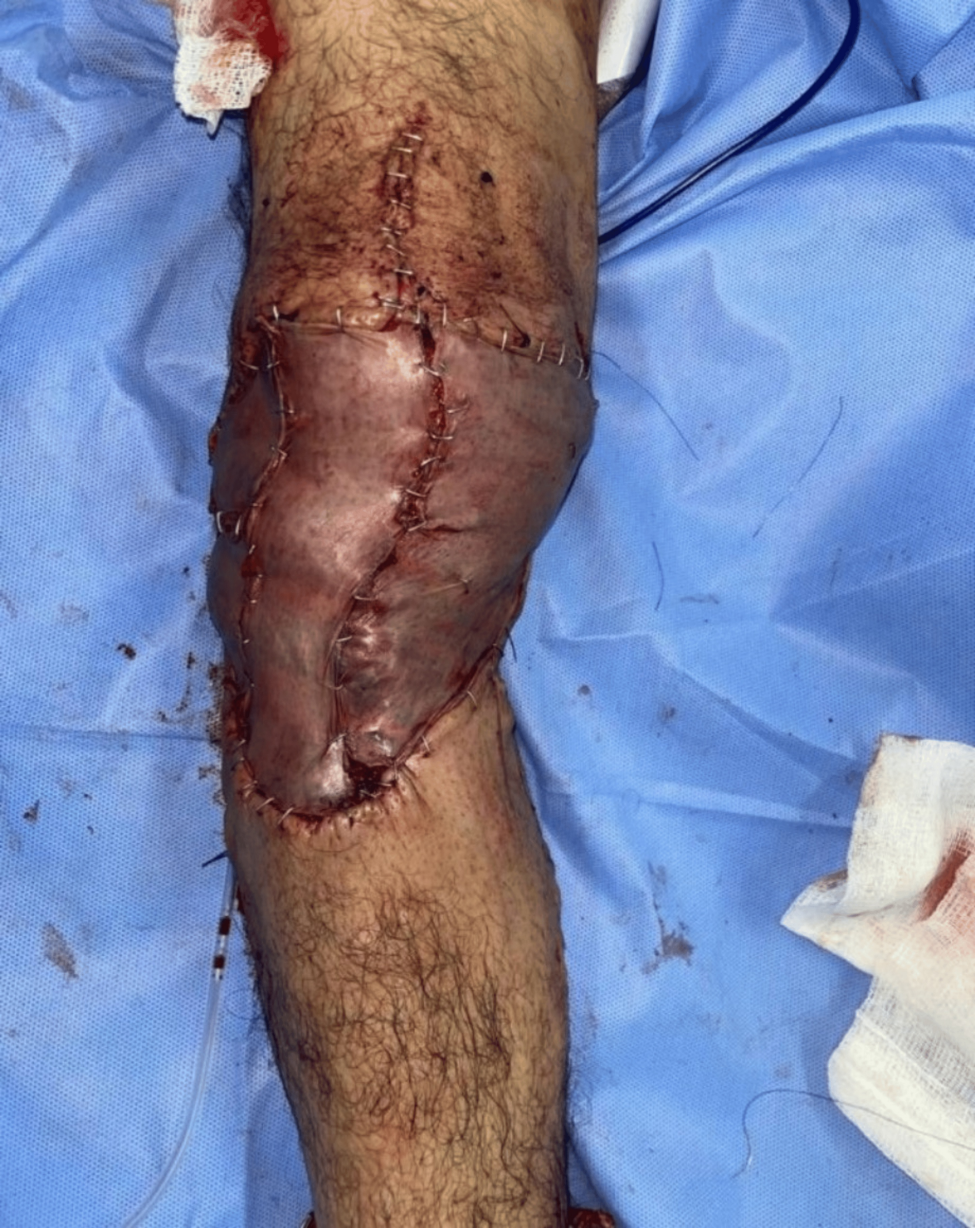
Surgical image showing the patient's skin coverage

For the reconstruction of the extensor mechanism, we identified and harvested the semitendinosus and gracilis tendons using a tendon stripper. We then created a transtibial tunnel and a transpatellar tunnel. A portion of the quadriceps tendon was harvested and fashioned into a Y-shaped graft to reinforce the construct. Finally, the quadriceps tendon was sutured to the gracilis and semitendinosus tendons using simple stitches, with additional reinforcement of the patellar tendon framework using a steel wire. Skin coverage was achieved through a gastrocnemius rotational flap and a skin graft, both performed by the plastic surgery team (Figures 7-8).

The postoperative course was uneventful, with no complications or pain. Histological examination of the surgical specimen confirmed a grade III myxoinflammatory fibroblastic sarcoma according to the FNCLCC classification, with clear and adequate resection margins. Immunohistochemical staining for Myogenin demonstrated positive expression, supporting the diagnosis of high-grade (Grade III) sarcoma with myogenic differentiation. CD68 immunostaining was positive in tumor cells. The bone tissue was free of tumor infiltration. The patient was referred to his oncologist for ongoing follow-up and further treatment. At the four-month follow-up, no local or distant recurrence was observed, with good wound healing and satisfactory functional recovery.

## Discussion

MIFS is an uncommon, low-grade malignant tumor of the soft tissues, first described in 1998 by Montgomery et al. and Michal et al. under varying nomenclatures [5,6]. Since its initial description, MIFS has remained a rare entity with limited awareness among clinicians and pathologists, often resulting in delayed diagnosis or misdiagnosis. It typically presents as a slow-growing, painless mass located in the distal extremities, with sizes ranging from small nodules to large, infiltrative lesions exceeding 10 cm [4,5].

Histologically, MIFS is characterized by a complex mixture of features, including bizarre pleomorphic spindle or epithelioid cells, pseudolipoblasts, and areas of myxoid stroma intermixed with dense inflammatory infiltrates [6]. This morphological heterogeneity, compounded by the presence of an inflammatory background, can easily mimic other benign or malignant processes, including pleomorphic sarcoma, inflammatory myxoid tumors, or reactive lesions.

The tumor is locally aggressive but rarely metastasizes distantly. It may be mistaken for an inflammatory process and remain untreated for extended periods. The lesion’s heterogeneity and the abundant inflammatory component can complicate the diagnosis for pathologists [6]. In our clinical case, the tumor’s location around the knee could easily be confused with bursitis or arthritis. A systematic and methodical diagnostic approach is essential. Although trauma may draw attention to the lesion, it does not play a role in tumor genesis [7].

Recent guidelines recommend that any suspicious swelling larger than 5 cm undergo magnetic resonance imaging (MRI) with contrast enhancement, followed by a needle biopsy [8].

Management of localized myxoinflammatory fibroblastic sarcoma relies on wide surgical excision with negative margins. The extent of margins depends on tumor site, grade, and planned or administered adjuvant treatments. When negative margins cannot be achieved, amputation or radiotherapy may be considered. Postoperative follow-up should include clinical and MRI evaluations for at least two to three years [9,10].

Oncologic surgery for myxoinflammatory fibroblastic sarcoma in a limb can threaten limb integrity. Plastic surgery, employing various reconstructive techniques, plays a crucial role in preserving limb function. In our case, we reconstructed the patellar tendon concurrently with tumor resection, although some surgeons prefer delayed reconstruction after tumor excision [11,12].

In the present case, the patient developed a progressively enlarging, painful mass of the right knee over three months, accompanied by restricted mobility. While the location is atypical for MIFS, which more commonly involves distal extremities, the clinical suspicion for a sarcomatous lesion was raised following imaging studies, which revealed a heterogeneous soft tissue mass with necrotic areas. Biopsy confirmed a high-grade pleomorphic sarcoma (FNCLCC Grade III), but the definitive diagnosis of myxoinflammatory fibroblastic sarcoma was made only after examination of the entire surgical specimen.

This discrepancy between the initial biopsy and the final diagnosis reflects a well-recognized diagnostic challenge in MIFS. The tumor is morphologically heterogeneous and often contains a mix of spindle cells, bizarre giant cells, and pseudolipoblasts in a myxoid or hyaline stroma, along with a dense inflammatory infiltrate. Small biopsy samples may not capture this full histological diversity, particularly in high-grade or pleomorphic variants, as seen in our case.

Diagnostic rationale and immunohistochemical considerations

One of the key findings in our case was the nuclear positivity for Myogenin, a transcription factor typically used as a sensitive and specific marker for rhabdomyoblastic differentiation. This result initially raised the differential diagnosis of rhabdomyosarcoma, particularly given the high-grade cytological features. However, several contextual and histopathologic factors led us to favor the diagnosis of high-grade MIFS with aberrant myogenic marker expression, rather than reclassifying the tumor entirely.

First, Myogenin positivity was focal, rather than diffuse, and lacked the supporting morphological features of rhabdomyosarcoma such as strap cells, eosinophilic cytoplasm, and cross-striations. Second, the overall architecture and cellular composition of the tumor, including the inflammatory background, myxoid zones, and the presence of bizarre and vacuolated tumor cells, were more in keeping with MIFS. Third, CD68 expression, a marker often expressed in MIFS and suggestive of histiocytic or fibroblastic differentiation, was also positive in tumor cells. This finding further supports a diagnosis of MIFS rather than a purely myogenic neoplasm.

While Myogenin expression is uncommon in MIFS, rare cases have been described in the literature showing atypical immunoprofiles, especially in high-grade tumors with increased pleomorphism. Our case contributes to this growing evidence that focal expression of myogenic markers may occur in MIFS, representing aberrant or divergent differentiation rather than a distinct line of histogenesis.

Thus, the final diagnosis of grade III MIFS with focal myogenic differentiation was based on an integrated approach, combining histopathologic examination of the full surgical specimen, immunohistochemical analysis, and clinical correlation.

Management and follow-up

Surgical management of MIFS typically involves wide excision with negative margins, given its high rate of local recurrence and rare metastatic potential [9,10]. In our case, the tumor required an extended resection, including partial patellectomy and excision of adjacent tendinous and bony structures. To preserve limb function, an immediate reconstructive procedure was performed using a combination of gracilis and semitendinosus tendons, a Y-shaped quadriceps graft, and a gastrocnemius rotational flap for soft tissue coverage. This multidisciplinary approach allowed for satisfactory oncologic and functional outcomes.

Postoperative histological examination confirmed complete excision with clear margins, absence of bone invasion, and FNCLCC Grade III status. At the four-month follow-up, there was no clinical or radiologic evidence of recurrence, and the patient had recovered good functional use of the limb.

This case highlights the diagnostic complexity of high-grade MIFS, particularly when presenting with unusual immunohistochemical profiles, such as Myogenin positivity. It underscores the importance of evaluating the entire tumor architecture and integrating histological findings with immunophenotypic and clinical data to avoid misclassification. Awareness of these atypical presentations is crucial for accurate diagnosis, appropriate treatment planning, and optimal patient outcomes.

 Table 2 presents the differences between the two histological types. 

**Table 2:** Distinction between undifferentiated pleomorphic sarcoma and myxoinflammatory fibroblastic sarcoma

	Undifferentiated Pleomorphic Sarcoma	Myxoinflammatory Fibroblastic Sarcoma
Typical Location	Deep soft tissues of the extremities and retroperitoneum	Distal extremities
Origin	Originates from primitive mesenchymal cells. These cells lack a specific line of differentiation, meaning they don't clearly belong to a particular tissue type.	Connective tissue, specifically the fibroblasts in the subcutaneous tissues
Patient Demographics	Predominantly affects older adults, with a peak incidence between the ages of 50 and 70	Affects adults, with a median age of around 40 years
Clinical Behavior	High-grade, aggressive, frequent metastasis	Low-grade, locally aggressive, rare metastasis
Appearance	Large, infiltrative, fleshy masses	Poorly circumscribed, multinodular, often gelatinous
Histological Features	Characterized by a marked pleomorphism of tumor cells, meaning they vary greatly in size and shape. These cells, often spindle-shaped, are arranged in a haphazard, storiform, or fascicular pattern with a high mitotic index.	Histologically, these tumors display a combination of features, including a low-grade-appearing spindle or epithelioid cell proliferation admixed with areas containing abundant myxoid stroma and prominent inflammatory elements. The most distinctive histologic feature, however, is the presence of scattered large, atypical cells that can exhibit marked nuclear pleomorphism and contain one or more prominent, inclusion-like nucleoli, sometimes resembling Reed–Sternberg cells or lipoblasts.
Necrosis	Frequent	Rare
Inflammatory Component	Usually absent	Prominent
Immunohistochemistry	Non-specific: may be positive for Vimentin and CD68	Variable: may be positive for Vimentin and CD68, MUC4, and CD163
Molecular Findings	Characterized by a complex karyotype with extensive genomic rearrangements and numerous copy number alterations	Several genetic alterations have been identified in MIFS, including an unbalanced t(1;10)(p22;q24) translocation that leads to juxtaposition of the TGFBR3 and OGA (formerly known as MGEA5) genes resulting in upregulation of NPM3 and FGF8, the formation of supernumerary ring chromosomes with an amplified region in chromosome 3 leading to overexpression of VGLL3, BRAF translocations involving ROBO1 and TOM1L2, and BRAF amplification.
Recurrence/Metastasis	High local recurrence and metastatic potential	High local recurrence and low metastatic risk
Treatment	Radical surgery with adjuvant chemotherapy and radiotherapy for unresectable tumors	Wide local excision and re-resection of recurrences

## Conclusions

Myxoinflammatory fibroblastic sarcoma is a rare soft tissue tumor predominantly affecting the extremities. It is a low-grade malignancy with minimal metastatic potential but a high likelihood of local recurrence. Prognosis largely depends on the timeliness of diagnosis and the adequacy of the initial oncological surgical treatment, which should aim to achieve clear margins while preserving function. Radiotherapy may be considered when necessary. A multidisciplinary approach is essential for optimal management of this tumor.
